# Rosette formation by *Plasmodium vivax* gametocytes favors the infection in *Anopheles aquasalis*

**DOI:** 10.3389/fcimb.2023.1108348

**Published:** 2023-02-15

**Authors:** Luis Carlos Salazar Alvarez, Vanessa Carneiro Barbosa, Omaira Vera Lizcano, Djane Clarys Baia da Silva, Rosa Amélia Gonçalves Santana, Camila Fabbri, Paulo Filemon Paoluci Pimenta, Wuelton Marcelo Monteiro, Letusa Albrecht, Marcus Vinicius Guimarães de Lacerda, Fabio Trindade Maranhão Costa, Stefanie Costa Pinto Lopes

**Affiliations:** ^1^ Centro Internacional de Pesquisa Clínica em Malária – CIPCliM, Fundação de Medicina Tropical - Dr. Heitor Vieira Dourado (FMT-HVD), Manaus, Brazil; ^2^ Programa de Pós-graduação em Medicina Tropical, Universidade do Estado do Amazonas, Manaus, Brazil; ^3^ Laboratory of Tropical Diseases-Prof. Dr. Luiz Jacintho da Silva, Department of Genetics, Evolution, Microbiology and Immunology, University of Campinas-UNICAMP, Campinas, Brazil; ^4^ Instituto Leônidas & Maria Deane (ILMD/Fiocruz Amazônia), Fundação Oswaldo Cruz (FIOCRUZ), Manaus, Brazil; ^5^ Grupo de investigación en Química y Biotecnología (QUIBIO), Facultad de Ciencias Básicas, Universidad Santiago de Cali, Cali, Colombia; ^6^ Departamento de Saúde Coletiva, Universidade Federal do Amazonas, Manaus, Brazil; ^7^ Universidade Nilton Lins, Manaus, Brazil; ^8^ Instituto de Pesquisas René Rachou (IRR/ Fiocruz Minas), FIOCRUZ, Belo Horizonte, Brazil; ^9^ Instituto Carlos Chagas (ICC/ Fiocruz Paraná), FIOCRUZ, Curitiba, Brazil

**Keywords:** malaria, gametocyte, rosetting, cytoadhesion, *Plasmodium vivax*, vector, anopheles

## Abstract

*Plasmodium vivax is* a public health problem and the most common type of malaria outside sub-Saharan Africa. The capacity of cytoadhesion, rosetting, and liver latent phase development could impact treatment and disease control. Although the ability to *P. vivax* gametocyte develop rosetting is known, it is not yet clear which role it plays during the infection and transmission process to the mosquito. Here, we used *ex vivo* approaches for evaluate the rosetting *P. vivax* gametocytes capacity and we have investigated the effect of this adhesive phenotype on the infection process in the vector *Anopheles aquasalis* mosquito. Rosette assays were performed in 107 isolates, and we have observed an elevated frequency of cytoadhesive phenomena (77,6%). The isolates with more than 10% of rosettes have presented a higher infection rate in *Anopheles aquasalis* (p=0.0252). Moreover, we found a positive correlation between the frequency of parasites in rosetting with the infection rate (p=0.0017) and intensity (p=0.0387) in the mosquito. The disruption of *P. vivax* rosette formation through mechanical rupture assay confirmed the previously findings, since the paired comparison showed that isolates with disrupted rosettes have a lower infection rate (p<0.0001) and intensity (p=0.0003) compared to the control group (no disruption). Herein we have demonstrated for the first time a potential effect of the rosette phenomenon on the infection process in the mosquito vector *An. aquasalis*, favoring its capacity and intensity of infection, thus allowing the perpetuation of the parasite cycle life.

## Introduction

Despite efforts to control malaria, this disease remains a public health problem. In 2021 were estimated more than 247 million cases and 619,000 deaths worldwide (WHO, 2022). Although malaria in humans is caused by six species (*P. falciparum*, *P. vivax*, *P. ovale*, *P. malariae*, *P. knowlesi* and *P. cynomolgi*), two are responsible for the most significant impact of this disease in the world. *P. falciparum* is responsible for the highest-burden of infections and deaths globally, followed by infections of *P. vivax*, the most geographically widespread malaria species (Battle et al., 2019; Antonelli et al., 2020).

In *Plasmodium* spp. infection, red blood cells (RBCs) could cointain parasite´s asexual mature forms or gametocytes which may be sequestered in the microvasculature of vital organs and can present the vascular endothelium attaching ability (cytoadherence) or aggregation of uninfected red blood cells (rosetting) (Chakravorty et al., 2008; Carvalho et al., 2010; Heussler et al., 2017; Salazar Alvarez et al., 2021). The rosette phenomenon has been observed in *P. falciparum*, *P. vivax*, and *P. ovale* infections (Wahlgren et al., 1994; Udomsanpetch et al., 1995; Angus et al., 1996) and are currently known to be formed by trophozoites, schizonts and gametocytes (Chotivanich et al., 1998). In *P. falciparum* infection, rosetting is associated with severe malaria (Ho et al., 1991; Rowe et al., 1995). However, this phenomenon is more frequently observed for *P. vivax* than *P. falciparum* infections (Chotivanich et al., 1998) and for the late has been commonly observed in non-severe malaria (Lee et al., 2014), although its specific role in its pathophysiology is unknown.

Even though the capacity of rosetting development in *P. vivax* infection has been described more than two decades ago, little information is found about this cytoadhesive phenomenon (Udomsanpetch et al., 1995). It has been shown that the receptors traditionally involved in the rosetting process in *P. falciparum* infections, such as the ABO and CD35 antigens, do not participate in the formation of rosettes in *P. vivax-infected* erythrocytes (Rowe et al., 1997; Barragan et al., 2000; Rowe et al., 2007; Vigan-Womas et al., 2012). Rosette formation in *P. vivax* seems related to the parasite and the glycophorin C (CD236R) receptor in erythrocytes (Lee et al., 2014). Furthermore, it has been shown that in *P. vivax* infections, rosette stability is not affected by shear stress *in vitro*, suggesting that rosetting is not affected by hemodynamic forces of *in vivo* circulation (Zhang et al., 2016). Recently, it has been shown that rosetting is possibly associated with immune evasion strategy (Albrecht et al., 2020). Lee et al. (2020) observed that when *P. vivax* or *P. falciparum* infected erythrocytes are exposed to leukocytes, the rosetting rate increased by insulin growth factor binding protein 7 (IGFBP7), leading to phagocytosis reduction (Lee et al., 2020). Furthermore, it has also been observed that *P. falciparum* rosetting can protect schizont from artemisinin treatment (Lee et al., 2021). Thus, it is clear that this cytoadhesive phenotype is an essential adaptation for the parasite survival during the life cycle in the human host; however, it is unclear what is the relationship of rosettes in gametogenesis, even in falciparum malaria, and if this phenomenon has any implication during the parasite transmission process from the human to the mosquito vector.

Experimental studies with *Leishmania* spp. showed that agglomeration between the parasites and host erythrocytes causes a limitation of the parasite exposure to the digestive enzymes of the sand fly’s midgut, leading to the parasite protection in early development stages within the insect vector (Pimenta et al., 1997). It has recently been shown that lower red blood cell concentration decreases *P. vivax* establishment of infection ability in *An. aquasalis*, indicating that the high RBC concentrations protect *P. vivax* from killing (Baia-da-Silva et al., 2018). These facts, associated with the ability of *P. vivax* (Pv-pRBC) infected erythrocytes to form rosettes, lead to the hypothesis of the possible role of rosettes in *P. vivax* gametocytes transmission, helping the parasite survival in the mosquito early stages of development. Here, we evaluate through *ex vivo* approaches the ability of gametocytes from non-severe *P. vivax* isolates to rosetting. In addition, we assessed whether this cytoadhesive phenomenon could contribute to the *An. aquasalis* mosquito infection.

## Materials and methods

### Sample collection

Blood samples were collected at the Fundação de Medicina Tropical Dr. Heitor Vieira Dourado (FMT-HVD), located in the city of Manaus, Amazonas state, Brazil. 107 adults’ patients (aged >18 years) with *P. vivax* mono-infection, microscopically diagnosed with parasitemia higher than or equal 1,000 parasites/µL and no current antimalarial treatment were included in the study. Before the treatment, a sample of 9 mL of peripheral blood was withdrawn using heparin-coated vacutainer tubes. Patients were treated following the Brazilian Health Ministry malaria treatment guidelines (Ministério da Saúde do Brasil, 2021).

### *Anopheles aquasalis* colony

*An. aquasalis* were reared in a well-established colony in the Medical Entomology Laboratory at FMT-HVD. Colonies were kept at 24–26 °C and relative humidity of 70–80% on a 12:12 light-dark cycle. Larvae were hatched at room temperature in water containing salt at a final concentration of 2g/L, and a fish food TetraMin^®^ (Madison, Wisconsin, USA) was provided daily. Larvae were allowed to pupate and become adults in an enclosed mesh-covered cage with water provided and fed ad libitum with a 10% sucrose solution until two days before being given the infective blood meals (Baia-da-Silva et al., 2018).

### *P. vivax* gametocyte purification

An aliquot of total blood was reserved to direct membrane feeding assay (DMFA) (control group). The percentage of *P. vivax* gametocytes-were determined by counting the blood smears stained with Giemsa. The blood was immediately processed to obtain enriched *P. vivax* - parasitized red blood cells (Pv-pRBC). Blood was centrifuged, separating plasma from RBCs. Plasma was stored at 4°C for rosette assay. The RBC were washed twice with RPMI-1640 medium (Sigma-Aldrich^®^, USA) and filtered in a cellulose column (Sigma-Aldrich^®^, USA) to remove leukocytes and platelets as previously described (Russell et al., 2011). A Percoll (GE Healthcare, USA) cushion (45%) was performed to separate the mature asexual parasites and gametocytes from the younger asexual forms and non-infected erythrocytes as previously described (Vera et al., 2015). The total number of gametocytes (gPv-pRBC) after the purification process was determined by counting the percentage of gametocytes in thin blood smears from the Percoll interface and determining the quantity of cells per ml using a Neubauer Chamber.

### Rosetting assay

In parallel to the DMFA (next section) gPv-pRBC were adjusted to 10% parasitemia and 5% hematocrit in a rosetting medium (McCoy5A medium supplemented with 20% native autologous plasma); after that, 50 µL of this suspension was plated in a 96 well plate (u- bottom), followed by incubation for 40 minutes at 37°C. Then, they were stained with 50µL of acridine orange (50 µg/mL) and incubated for 10 minutes at 37°C. Subsequently, one drop of the stained suspension was pipetted onto a glass slide and immediately covered with a glass cover slip. Finally, the rosette formation was examined by direct light and fluorescence microscopy (Nikon Eclipse 50i, filter 96311 B-2E/C) (Udomsanpetch et al., 1995). The proportion of gPv-pRBC in rosettes was measured by counting 100 gPv-pRBC in each experiment in duplicate. A rosette was determined by the adhesion of two or more uninfected erythrocytes to a gPv-pRBC (**Supplementary Figure S1**). Giant erythrocyte rosettes surrounding a *P. vivax* infected cell were also counted (Marin-Menendez et al., 2013). The isolates were classified into rosette (rosette formation rate higher than or equal to 10%) and non-rosette-forming (rosette forming rate less than 10%).

### Disrupted rosettes assays

After the incubation to evaluate the rosette formation, the cell suspension from 4 wells of the 96 well plates was collected in a 1.5 mL microtube per group (200 µL in total). One microtube were maintained in static condition at room temperature (intact rosettes) and the other was exposed to constant vortex agitation for 5 min after completely disrupting rosettes using a needle (disrupted rosettes). Subsequently, membrane feeding assay was performed with these samples (intact and disrupted rosettes) following the parameters described in *P. vivax* DMFA.

### *P. vivax* DMFA

The DMFA was conducted according Fabbri et al., 2021 with few modifications. Three different sets of DMFA experiments were performed. The first one was performed in order to verify if the procedure of parasite purification interfere with *An. aquasalis* infection, and therefore DMFA were performed comparing two groups: total blood and gPv-pRBC. For this first set of experiments, 500 µL of the total blood were separated and maintained at 37 °C until be used in the DMFA. The remaining blood was processed for gametocyte purification as described above. The pellet of cells obtained was suspended in non-infected O-type blood and AB serum to a hematocrit of 40%. Both suspensions were given to fed mosquitos in the DMFA.

The second set of experiments was performed using 47 isolates enriched to gPv-pRBC to verify the impact of *P. vivax* rosette at *Anopheles* infection. For this, part of the purified sample was used to set the rosetting assay as described above and around 1x10^5^ gPv-pRBC were suspended in non-infect O-type blood and autologous plasma to a hematocrit of 40% and given to fed mosquitoes immediately.

The last set of experiments was performed to evaluate the rosette effect in *Anopheles* infection in the same isolate and for this purpose a mechanical rupture of rosettes were performed. Briefly, after the identification of positive samples for the rosette phenomenon (rosette formation rate higher than or equal to 10%), these were divided into two aliquots for mosquito feeding. One aliquot was kept at room temperature in static conditions (intact rosettes), and the other was subjected to mechanical rupture (disrupted rosettes). After that, the samples were centrifuged and suspended in non-infect O-type blood and autologous plasma to a hematocrit of 40% and given to fed mosquitoes immediately.

The blood suspension in different conditions was offered to groups of 120-150 *An. aquasalis* females between 90-120 minutes through membrane feeders at 37 °C. After the blood-feeding experiments, only fully engorged mosquitoes were transferred to rearing containers; they were maintained in the insectary at 26 °C, 70–80% relative humidity, and fed daily on a 10% sugar solution. Seven days post-infection, the surviving mosquitoes from each experimental group were dissected. The midguts were stained with 2% commercial Mercurochrome (Merbromin, Sigma- Aldrich, USA), placed under a cover glass, and examined for the presence of oocysts in an optical microscope. The infection rate (percentage of infected mosquito midguts) and the infection intensity (mean number of oocysts per midgut) on each mosquito were recorded and compared among the experimental groups.

### Statistical analysis

Shapiro-Wilk test was evaluated to verify the normality of the distribution. A non-parametric Kruskal-Wallis test or Wilcoxon test were used for infection intensity analysis and for the infection rate, Mann Whitney parametric distribution. Correlations were analyzed using Pearson test. Statistical significance was defined as p < 0.05. All statistical analyses were conducted using GraphPad Prism version 8 software (GraphPad Software, Inc., San Diego, CA). The statistical tests applied are indicated in the figure’s caption, and (n) means the number of samples analyzed.

## Results

### *P. vivax* gametocytes’ capacity to form rosetting

**Figure 1:**
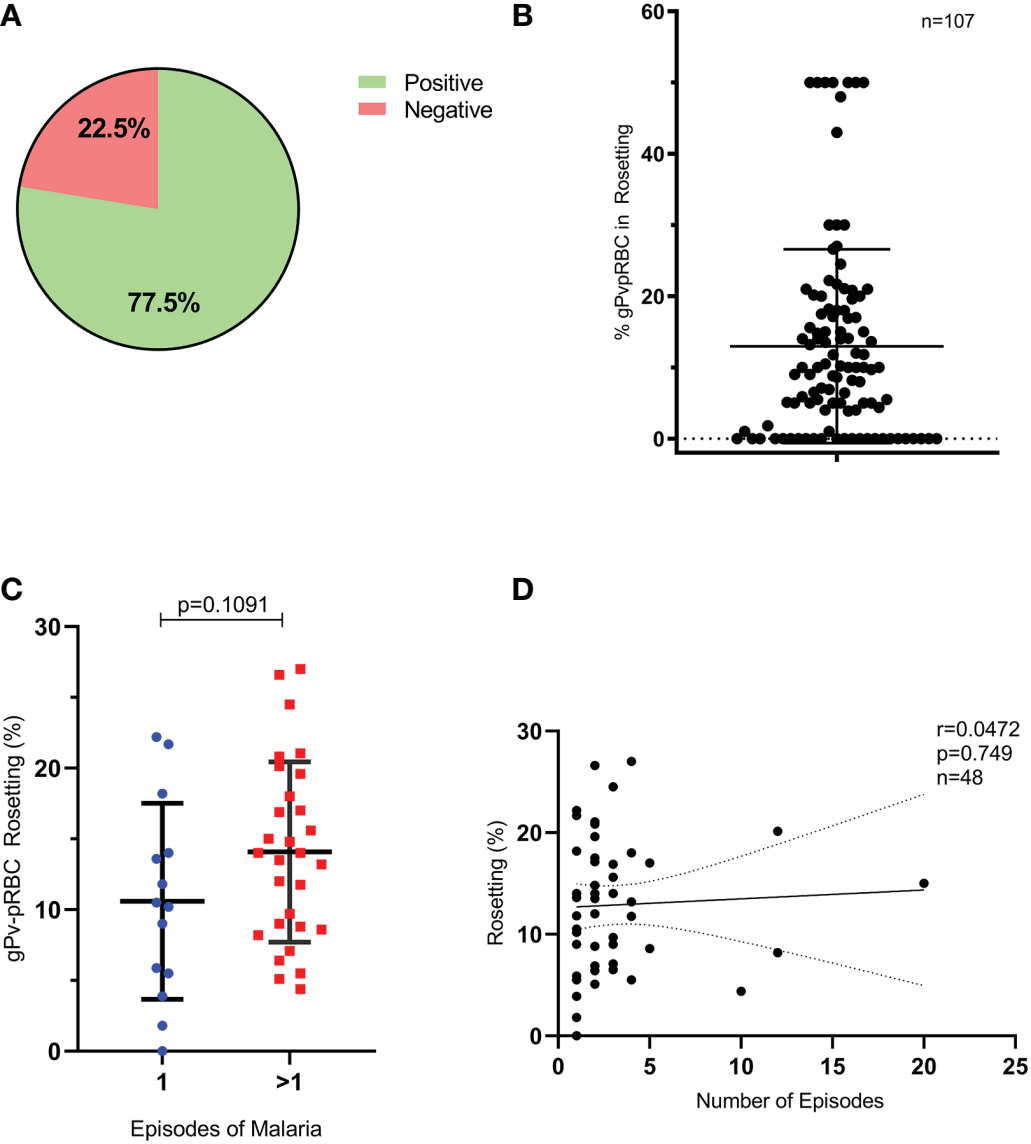
Formation of rosettes and previous episodes of malaria. **(A)** Samples tested with rosette formation capacity (n= 107). **(B)** Percentage of g*Pv*-pRBC with capacity of rosettes forming for each isolate (n=107). **(C)** gPv-pRBC rate forming rosettes in primoinfection and in multiple infections by *P. vivax.* **(D)** Correlation between the rate of gPv-pRBC in rosette formation and the number of previous episodes of malaria. Data are shown as the mean ± standard deviation. Rosetting rate among isolates was compared by unpaired-t test; p=0.1091; *n*=48.

The *P. vivax* gametocytes’ capacity to develop rosetting was assessed in 107 isolates obtained from patients with non-severe vivax malaria blood. The rosetting assay was developed with native autologous plasma, and 77.5% (83/107) of the tested samples presented rosette formation capacity (**Figure 1A**). The percentage of rosettes in gPv-pRBC in the samples evaluated presented a mean of 12.97% ± 13.61, ranging from 0 to 50% (**Figure 1B**). Additionally, we observed that the average of gPv-pRBC in rosette formation was higher in isolates from patients who had contracted malaria previously than in the primo infection ones (14.08 and 10.59% respectively); however, it was not statistically significant (Student’s t-test, *p* = 0.1091, n = 48) (**Figure 1C**). Moreover, no correlation between the number of previous episodes and rosettes rate was observed (Spearman’s test, p = 0.749; Pearson test) (**Figure 1D**). Finally, correlation analysis with the hematocrit, parasitemia and gametocytemia levels of the patients, showed no correlation with the rosetting capacity of the isolates analyzed in the present study. (**Supplementary Figures S2A–C**).

### gPv-pRBC rosettes formation and *Anopheles aquasalis* infectivity

**Figure 2:**
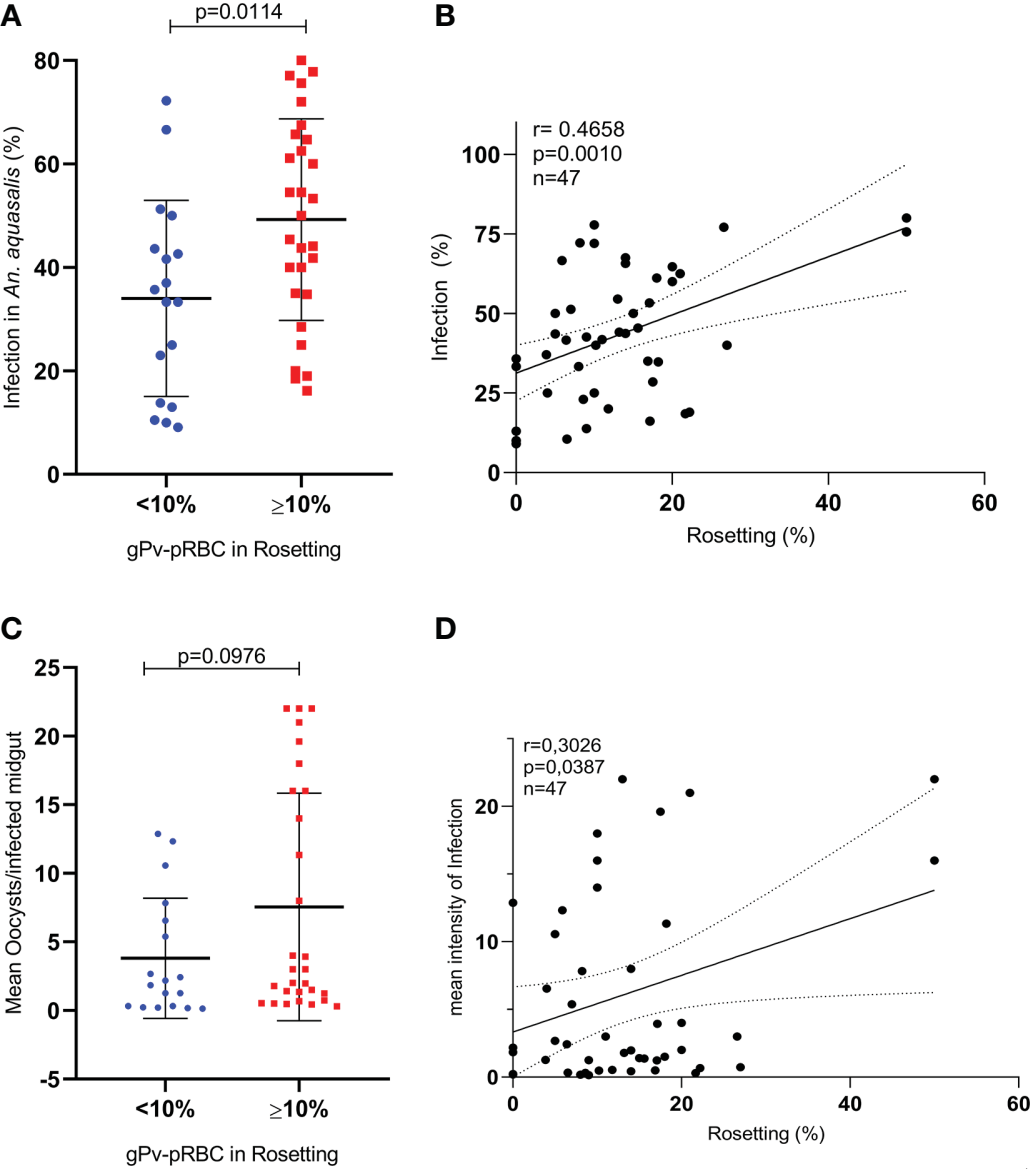
Infection of *Anopheles aquasalis* with g*Pv*RBC and rosette formation rates (DMFA (2)). **(A)** Infection rate of *An. aquasalis* with isolates of gPv-pRBC not rosette-forming (<10%; n=18) or rosette formers (>10%; n=29) **(B)** Correlation between the rate of gPv-pRBC in rosette formation and the infection rate in *An. aquasalis*. **(C)** Infection intensity of *An. aquasalis* with gPv-pRBC rosette formers (≥10%; n =29) and non-rosetters formers (<10%; n=18). **(D)** Correlation between the rate of gPv-pRBC in rosetting formation and the intensity of infection in *An. aquasalis*. The data are shown as the mean ± standard deviation from a total of 47 biological replicates (isolates). Isolation rate and intensity of infection were compared by t-test and Mann Whitney test, respectively. The correlational analyses were performed using the Spearman test.

Initially, we evaluated whether the purification process of gPv-pRBC could affect mosquito infectivity to verify if this process does not interfere with the parasites’ ability to infect the mosquitoes. No changes were observed in the *An. aquasalis* infection rate (p = 0.0986; Student’s t-test) and in the infection intensity (p = 0.5075; Mann Whitney test) (**Supplementary Figure S3**). Subsequently, among the 107 samples assessed for rosetting ability, 47 were used to evaluate the role of rosetting in *Anopheles aquasalis* infection by DMFA. Twenty-nine isolates of gPv-pRBC (61.7%) were rosette producers and showed 10 to 50% of rosette formation rates. In these isolates, infection rate was 49.25%, while the non-rosette forming isolates (n=18) had a 33.98% (p = 0.0114; Student’s t-test) (**Figure 2A**). Moreover, a positive correlation between the rosette and infection rate was found (p = 0.001; Pearson test) (**Figure 2B**). However, no difference between rosette forming and non-rosette forming group in infection intensity was observed (**Figure 2C**) but a positive correlation between the rosette rate and intensity of infection for these isolates was evidenced (p = 0.0387; Pearson test) ((**Figure 2D**).

### Integrity to gPv-pRBC rosetting correlates with capacity to *An. aquasalis* infectivity

**Figure 3:**
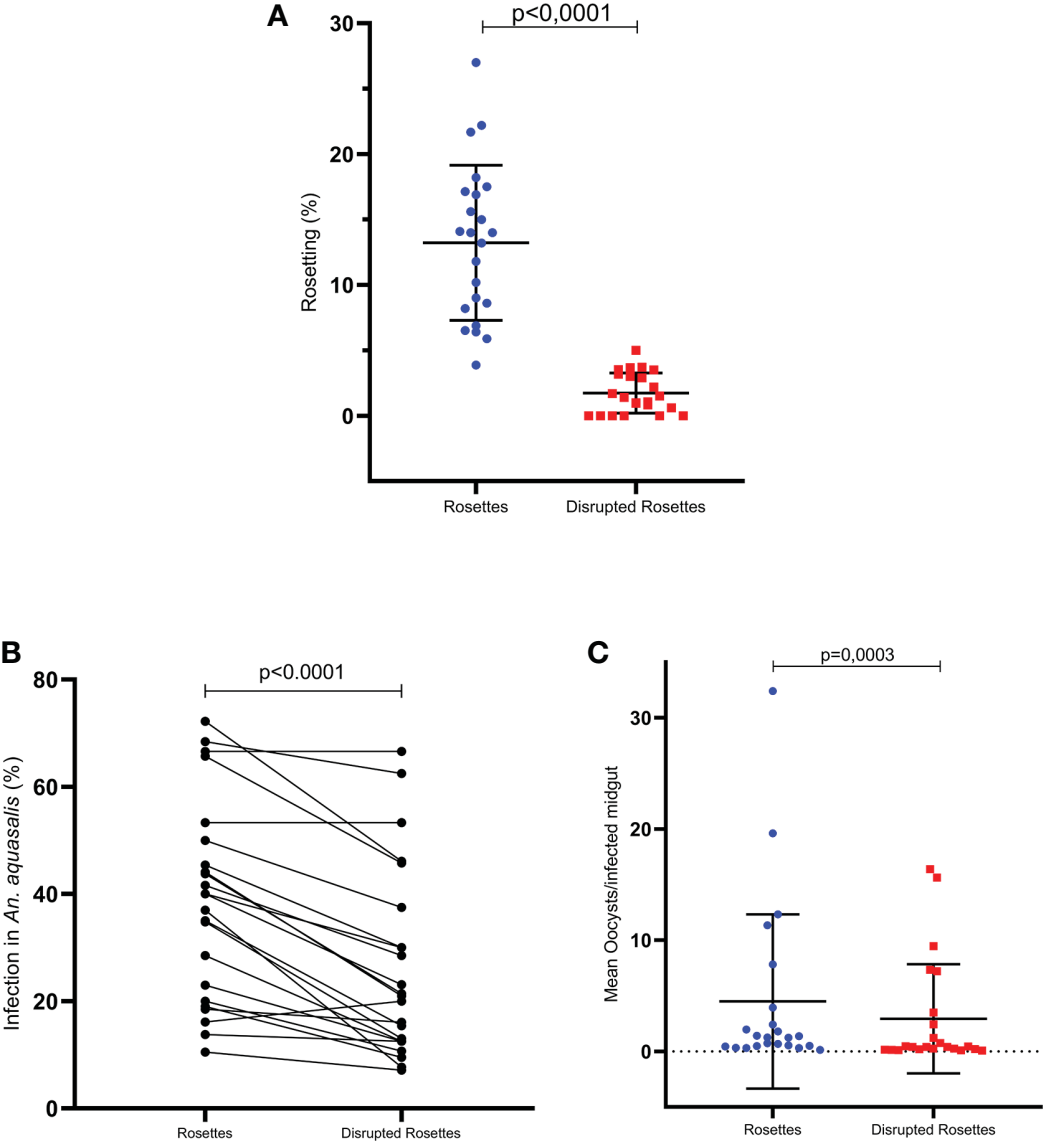
Integrity to gPv-pRBC rosetting correlates with *An. aquasalis* infectivity (DMFA (3)). **(A)** Effect to treatment to disrupted integrity to gPv-pRBC with capacity rosetting (n=23) **(B)** Infection rate of *An. aquasalis* with isolates of gPv-pRBC Rosette-forming *vs.* Disrupted Rosettes (n=23) **(C)** Infection intensity of *An. aquasalis* with isolates of gPv-pRBC Rosette-forming *vs.* Disrupted Rosettes (n=23). Data are shown as the mean ± standard deviation. The data are shown as the mean ± standard deviation from a total of 23 isolates. Isolation rate and intensity of infection before and after processing were compared by paired t-test and Wilcoxon test, respectively. The correlational analyses were performed using the Spearman test.

Once rosetting seems to be correlated with infection rate and intensity, 23 rosette rupture assays were performed. The mechanical rosette rupture process was effective, showing a decrease in rosette rate, which ranged from 0 to 5% after the procedure; and it was significantly different compared to the same untreated sample (p < 0.0001, Wilcoxon paired test) (**Figure 3A**). Therefore, the investigation of the ability to infect mosquitoes were performed by DMFA comparing the two groups: intact and disrupted rosettes. It was observed that all isolates assessed were able to infect *An. aquasalis*, presenting a mean infection rate of 37.6%, ranging from 7.14 to 72.2%, and a mean infection intensity of 28.67 ± 33.26, ranging from 0.30 to 32.42. The paired comparison between groups showed that the infection rate was higher in the intact (37.95 ± 19.20) than in the ruptured (26.23 ± 17.73) rosette groups (p < 0.0001; Wilcoxon test) (**Figure 3B**). Moreover, the infection intensity was also higher in the intact rosettes (4.49 ± 7.83) than in the rupture group (2.93 ± 4.91) (p= 0.0003; Wilcoxon paired test) (**Figure 3C**).

## Discussion

Rosetting is a cytoadhesive *Plasmodium* spp. phenotype that frequently occurs in *P. falciparum, P. vivax, and P. ovale* infections (David et al., 1988; Angus et al., 1996; Chotivanich et al., 1998). *P. vivax* present ability of rosettes formation in asexual and sexual parasites (Lee et al., 2014). In this study, we observed the high ability of *P. vivax* gametocytes to form rosettes in samples from non-severe vivax malaria, corroborating previous studies (Lee et al., 2014; Albrecht et al., 2020). Noteworthy, we have revealed the impact of rosette formation in mosquito infectivity.

Previous studies on the rosette formation phenomenon have been able to shed light on some factors involved in this phenotype at a molecular level, however the biological role of rosettes formation is not clear yet (Rowe et al., 1997; Chen et al., 1998; Barragan et al., 2000; Marti et al., 2014; Goel et al., 2015). According to some studies, the process of rosette is involved in parasite immune evasion strategy (Moll et al., 2015; Albrecht et al., 2020; Lee et al., 2020).

Although this phenomenon gives a biological advantage to the parasite, until then its role in the mosquito vector was not investigated. Herein, we demonstrated that rosette formation seems to be involved in an increase in the infectivity of the parasite to the mosquito since isolates that possess gPv-pRBC in rosette formation presented a higher rate and intensity infection in *An. aquasalis* compared to those who didn´t develop this phenotype. Recently, Zhang et al., 2016 showed that rosette formation in *P. vivax* infections is highly stable and not destroyed even under high pressure, leading us to hypothesize that rosettes are maintained during the mosquito blood-feeding. Furthermore, we found a positive correlation between the gPv-pRBC in rosettes rate and the infection intensity and rate, which directly impacts parasite transmission. Similarly, studies in *Leishmania* spp. (Pimenta et al., 1997) and *P. chabaudi* (Trager et al., 2005) have shown the relationship between blood cell density and parasite survival favors the early part of the infection on mosquito vector midgut.

Baia-da-Silva et al. (2018) have demonstrated that hematocrit affects *P. vivax* parasite in the *Anopheles aquasalis* midgut with lower hematocrit resulting in a lower infection in mosquitoes. The authors suggested that the observed may be due to the proximity of digestive enzymes, since the food bolus is small in the mosquitoes fed with low hematocrit. Similarly, our findings may be due to the protection of digestive enzymes by the non-infected erythrocytes around the infected one in the rosetting isolates.

Finally, we have move away the possibility of the rosettes effect in the increase in *P. vivax* infectivity in *An. aquasalis* observed be attributed to the genetic variation presented by the distinct phenotype isolates (rosette formers or non-formers) since we have demonstrated the same effect in a paired analysis using the same isolate exposed or not to mechanical rosetting rupture.

In conclusion, rosette phenomenon could protect the parasite from recognition by the immune system and the rosette formation seems to impact the transmission capacity of *P. vivax* by *An. Aquasalis* significantly. However, more studies should be carried out to comprehend the role of this phenomenon in parasite survival and transmission to the mosquito.

## Data availability statement

The original contributions presented in the study are included in the article/**Supplementary Material**. Further inquiries can be directed to the corresponding authors.

## Ethics statement

The studies involving human participants were reviewed and approved by the ethical board of Fundação de Medicina Tropical Dr. Heitor Vieira Dourado (CAAE: 54532416.0.0000.0005, approval number 1.591.958). The patients/participants provided their written informed consent to participate in this study.

## Author contributions

LCSA, VCB, SCPL and OVL performed P. vivax gametocytes purification, rosetting assay and sample organization. LCSA, OVL, CF, VCB were responsible for experimental infection and sample organization. LCSA, WMM and SCPL helped with statistical analysis. LCSA, LA, CF, DCBS, MVGL, PFPP, FTMC, WMM and SCPL helped draft the manuscript and manuscript revision. LCSA, LA, MVGL, FTMC, PFPP, WMM and SCPL participated in study design, coordination, and writing the finalversion of the manuscript. All authors read and approved the final manuscript.
