# Supplementary material for: Rosette formation by Plasmodium vivax gametocytes favors the infection in Anopheles aquasalis
Source: Front Cell Infect Microbiol. 2023 Feb 15;13:1108348. doi: 10.3389/fcimb.2023.1108348 (PMC9975573; doi:10.3389/fcimb.2023.1108348)
Supplement: Supplementary file 1 [file DataSheet_1.pdf]

Rosette formation by *Plasmodium vivax* gametocytes favors the infection in *Anopheles aquasalis*

Luis Carlos Salazar Alvarez[1,2,3#], Vanessa Carneiro Barbosa[1,4#], Omaira Vera Lizcano[1,3,5], Djane Clarys Baia da Silva[1,2,4,6,7], Rosa Amélia Gonçalves Santana[1,5], Camila Fabbri[1,2], Paulo Filemon Paoluci Pimenta[1,8], Wuelton Marcelo Monteiro[1,2], Letusa Albrecht [3,9], Marcus Vinicius Guimarães de Lacerda[1,5], Fabio Trindade Maranhão Costa[3*], Stefanie Costa Pinto Lopes[1,5*]

[1] Centro Internacional de Pesquisa Clínica em Malária – CIPCliM, Fundação de Medicina Tropical - Dr. Heitor Vieira Dourado (FMT-HVD), Manaus, Brazil;
[2] Programa de Pós-graduação em Medicina Tropical, Universidade do Estado do Amazonas, Manaus, Brazil;
[3]Laboratory of Tropical Diseases-Prof. Dr. Luiz Jacintho da Silva, Department of Genetics, Evolution, Microbiology and Immunology, University of Campinas-UNICAMP, Campinas, Brazil;
[4]Instituto Leônidas & Maria Deane (ILMD/Fiocruz Amazônia), Manaus, Brazil;
[5]Grupo de investigación en Química y Biotecnología (QUIBIO), Facultad de Ciencias Básicas, Universidad Santiago de Cali, Cali, Colombia.
[6]Universidade Federal do Amazonas, Manaus, Brazil;
[7]Universidade Nilton Lins, Manaus, Brazil;
[8]Instituto de Pesquisas René Rachou (IRR/ Fiocruz Minas), Belo Horizonte, Brazil;
[9]Instituto Carlos Chagas (ICC/ Fiocruz Paraná), Curitiba, Brazil.

Supplementary Information

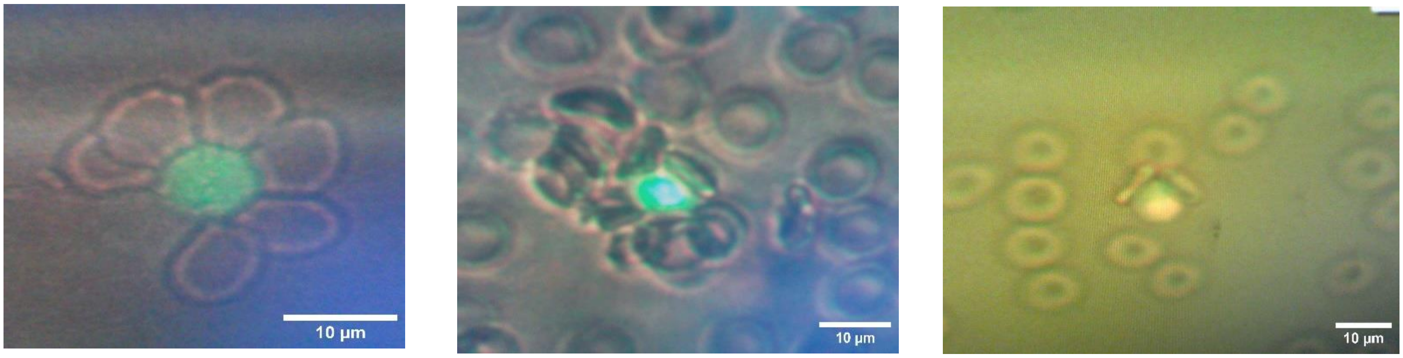

Supplementary Figure 1. Photomicrographs representatives of capacity to *P. vivax* formation rosetting.

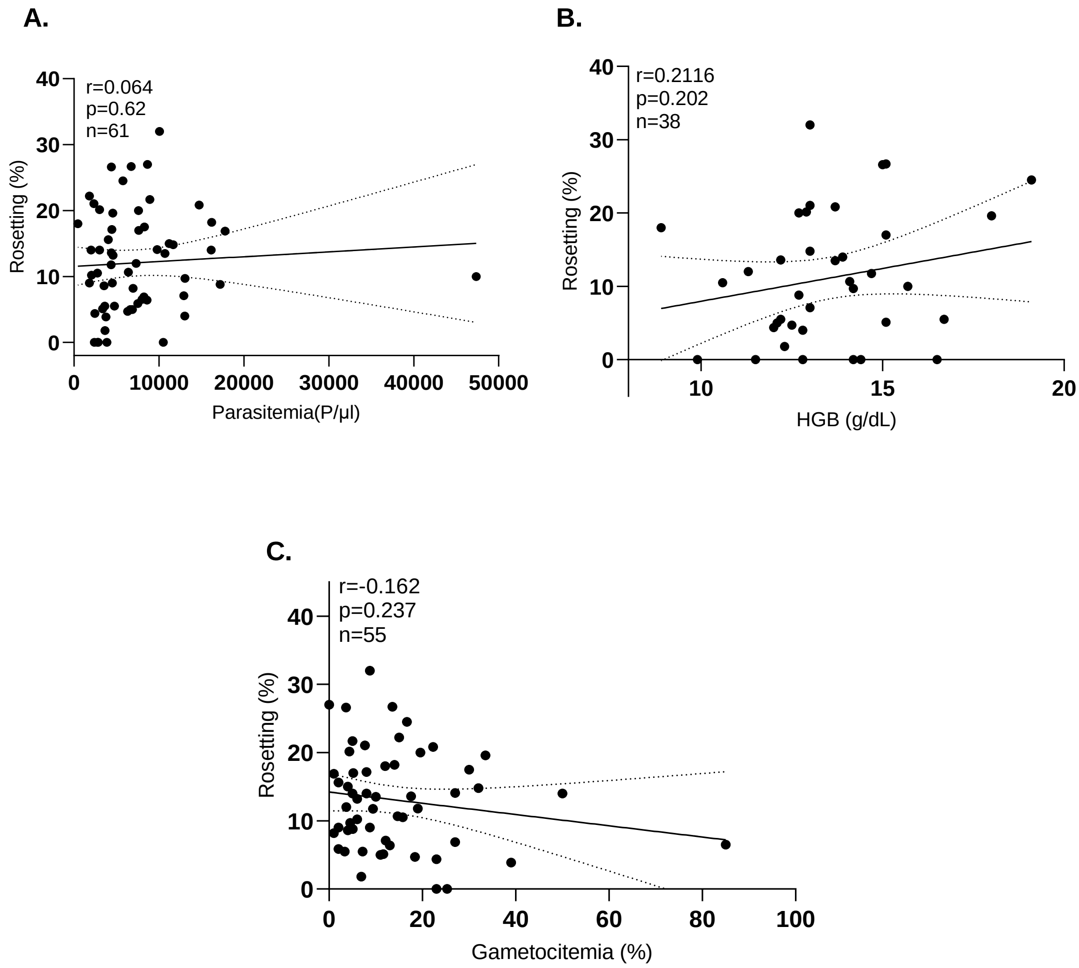

Supplementary Figure 2. A. Correlation analysis of the rosetting capacity and parasitaemia of the patients. **B**. Hematocrit level of the patients and their ability to rosetting. **C.** Correlation analysis of the Gametocythemia of the patient's isolate and its ability to rosetting.

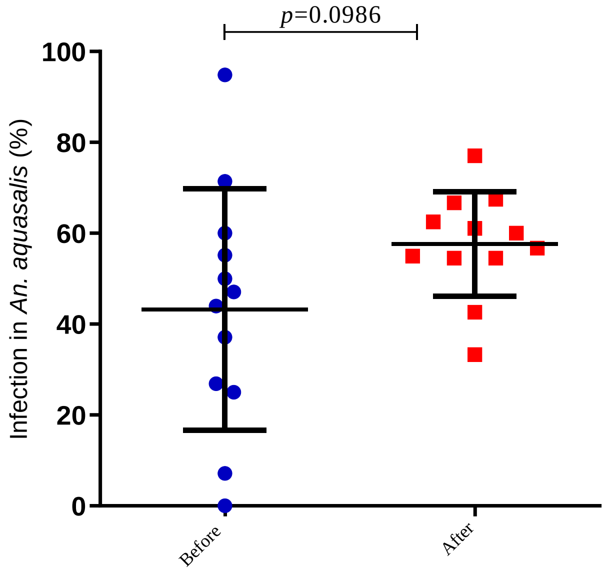

Supplementary Figure 3. A. Infection rate of *An. aquasalis* with gPv-pRBC before or after purification. The data are shown as the mean ± standard deviation from a total of 12 biological replicates (isolates). Isolation rate and intensity of infection before and after processing were compared by paired t-test and Wilcoxon test, respectively.
